# An evaluation of the occurrence and trends in ^137^Cs and ^40^K radioactivity in King Bolete *Boletus edulis* mushrooms in Poland during 1995–2019

**DOI:** 10.1007/s11356-021-12433-8

**Published:** 2021-02-24

**Authors:** Jerzy Falandysz, Tamara Zalewska, Michał Saniewski, Alwyn R. Fernandes

**Affiliations:** 1 University of Gdańsk, Environmental Chemistry and Ecotoxicology, 63 Wita Stwosza Street, 80-308 Gdańsk, Poland; 2 Institute of Meteorology and Water Management – Maritime Branch, National Research Institute, 42 Waszyngtona Av, 81-342 Gdynia, Poland; 3 School of Environmental Sciences, University of East Anglia, Norwich, NR4 7TJ UK

**Keywords:** Radiocaesium, Edible wild mushrooms, Human exposure, Organic food, Chernobyl fallout

## Abstract

*B. edulis*, collected from 33 forested or woodland sites across Poland over 25 years since 1995, were analysed for radiocaesium. The results (^137^Cs activity range: 25 to 10,000 Bq kg^-1^ dry weight) provide a good indication of artificial radioactivity in this food material. The relatively higher levels detected in the earlier years, mostly in easterly location, is consistent with depositions from the projected Chernobyl incident (1986) fallout plumes. Nevertheless, the ^137^Cs concentrations during 1995–2010 were, on average, higher than those reported by other studies for Polish *B. edulis* over the period 1986–1994. The data concurs with the general hypothesis and observations that deposited ^137^Cs permeates slowly over time to deeper soil horizons which host the mycelial networks. This delay in availability shows that (apart from hotspots) higher contamination of fruiting bodies occurred around 10 to 20 years after the incident. Local consumers and recreational mushroomers were undoubtedly exposed, although reported ^137^Cs concentrations suggest that serious breaches of regulated levels were uncommon.

## Introduction

Wild edible mushrooms are a valuable food resource that is appreciated in many regions of the world. A variety of species are collected, by foragers who depend on these fungi to complement their dietary requirements but also by recreational pickers who enjoy the pastime and the resulting mushroom meal. Some foragers with easy access to local forests and woodlands and their wild food resources also supplement their incomes by selling collected specimens by the road-side or at local outdoor markets. *Coprinus comatus* (shaggy mane or shaggy ink cap mushroom) is a common species that is found in a wide variety of habitats and is often seen in fields, lawns, grasslands and meadows. It is a versatile species and can also grow in urbanised, industrial areas and by the side of roads. On the other hand, matsutake (*Tricholoma matsutake*) mushrooms grow in specific forested terrains and are difficult to find. Their specific growth requirements make them rare, and foragers have to compete with wild animals such as deer and rabbits, making them highly prized (and expensive if sold), particularly in East Asian cuisine. For example, freshly collected *T. matsutake* can be shipped within a day or two, to several thousand kilometres away from their collection site, e.g. from the northern Yunnan in China to Japan, where they command high prices (Arora and Shepard 2008). There are a variety of preparation and cooking practices in different parts of the world depending on the seasonal availability of wild mushrooms, and fresh as well as preserved mushrooms are used in domestic cooking and in restaurants (usually as a seasonal food). Preserved wild mushrooms (dried, pickled, stir-fried or soured) are traded and available from shops as a delicacy or as functional foods, and they are usually more expensive than staple foods.

The situation with domesticated (cultivated) mushrooms is very different, and much depends on the cultivars that are used in production. These mushroom bear only a small sensory resemblance to the original wild species and, usually, only when fresh. The shitake mushroom (*Lentinula edodes*), when correctly cultivated using cedar wood, is an exception because it retains the original taste and properties of the wild specimens. For the general public, the taste of “mushrooms” is largely associated with the button mushroom (*Agaricus bisporus*), which is widely available, and to a smaller extent with the oyster mushroom (*Pleurotus ostreatus*), which curiously retains much of the taste of the wild species, in comparison to many other domesticated and cultured species which in sensory properties are a pale reflection of their wild counterparts.

The collection of mushrooms either by dependent or recreational pickers or by commercial foragers is a long lasting tradition with roots in the countryside rather than cities, and with a cultural heritage in many regions around the world. Since the twentieth century, the practice occurs away from densely populated areas and is more common in regions with preserved forests and woodlands, e.g. in Europe.

One of the negative aspects of consuming edible wild mushrooms is their propensity for accumulating contaminants such as heavy metals or radioactivity. The former has always been a risk particularly in areas where the local soils are naturally enriched with, e.g. mercury, or through anthropogenic activities involving metals or extracting metal ores. Local foragers are aware of historical risks and can take precautions with the rate of consumptions or through the use of other decontamination measures. Radioactivity, however, has become a more substantial risk to wild mushroom consumers in recent decades, since the testing and use of nuclear weapons and the wider use of nuclear fuel to generate electrical power.

**Fig. 1:**
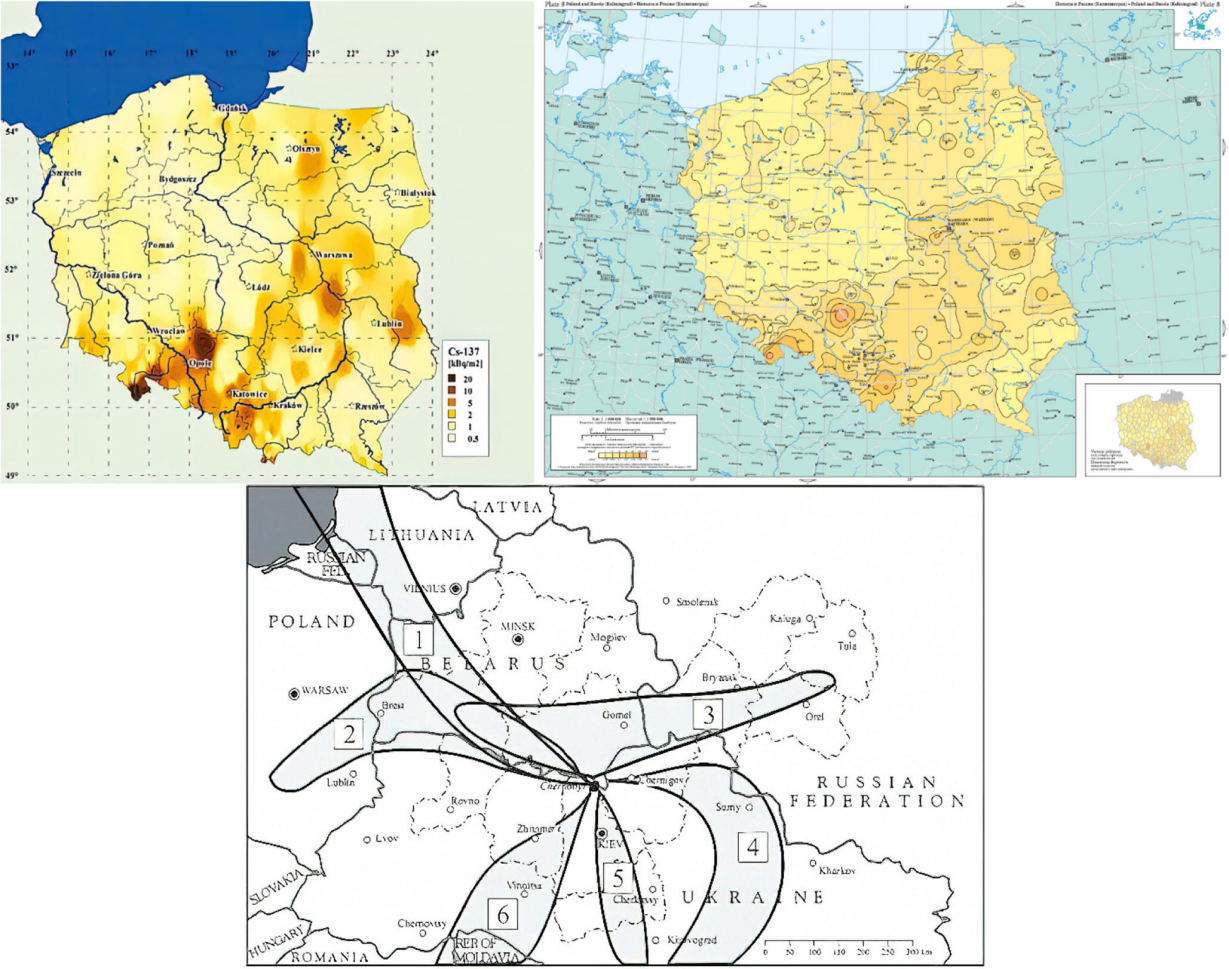
^137^Cs deposition in Poland showing affected areas and projected plume formations—numbers 1 to 6 refer to calculated plume formation according to the meteorological conditions for instantaneous releases on the following dates and times (Greenwich Mean Time): (1) 26 April 1986, 00:00; (2) 27 April, 00:00; (3) 27 April, 12:00; (4) 29 April, 00:00; (5) 2 May, 00:00; and (6) 4 May, 12:00. Illustrations taken from left (Isajenko et al. 2012), right (De Cort et al., 1998) and lower central (IAEA, 2006)

This evaluation examines the occurrence and history of pollution with radiocaesium (largely ^137^Cs) and naturally occurring potassium (^40^K) in one of the most prized and sought after wild mushroom species—the King Bolete or *B. edulis—*in Poland. This species is relatively abundant in Poland and is therefore the most frequently collected and exported mushroom species thus far. The study specimens were collected over a period of 25 years from 1995 to 2019 from wild habitats in Poland. Geographically and from the context of radioactive pollution, Poland neighbours the Ukraine and lies around 500 km west of the Chernobyl nuclear power plant, which suffered the worst known nuclear accident in history. Although Poland was not among the worst affected countries (Belarus, Russia and Ukraine itself suffered the worst impacts) (Grodzinskaya et al. 2003), its proximity resulted in some significant radioactive impacts in the form of contaminated air plumes from the stricken plant during the period of its collapse on April 26, 1986. The deposition rates of radiocaesium, due to plume waves and varying weather condition and differences in rainfall (fallout is enhanced during rainfall), showed some heterogeneous distribution and hotspots as shown in Fig. 1.

## Materials and methods

**Fig. 2:**
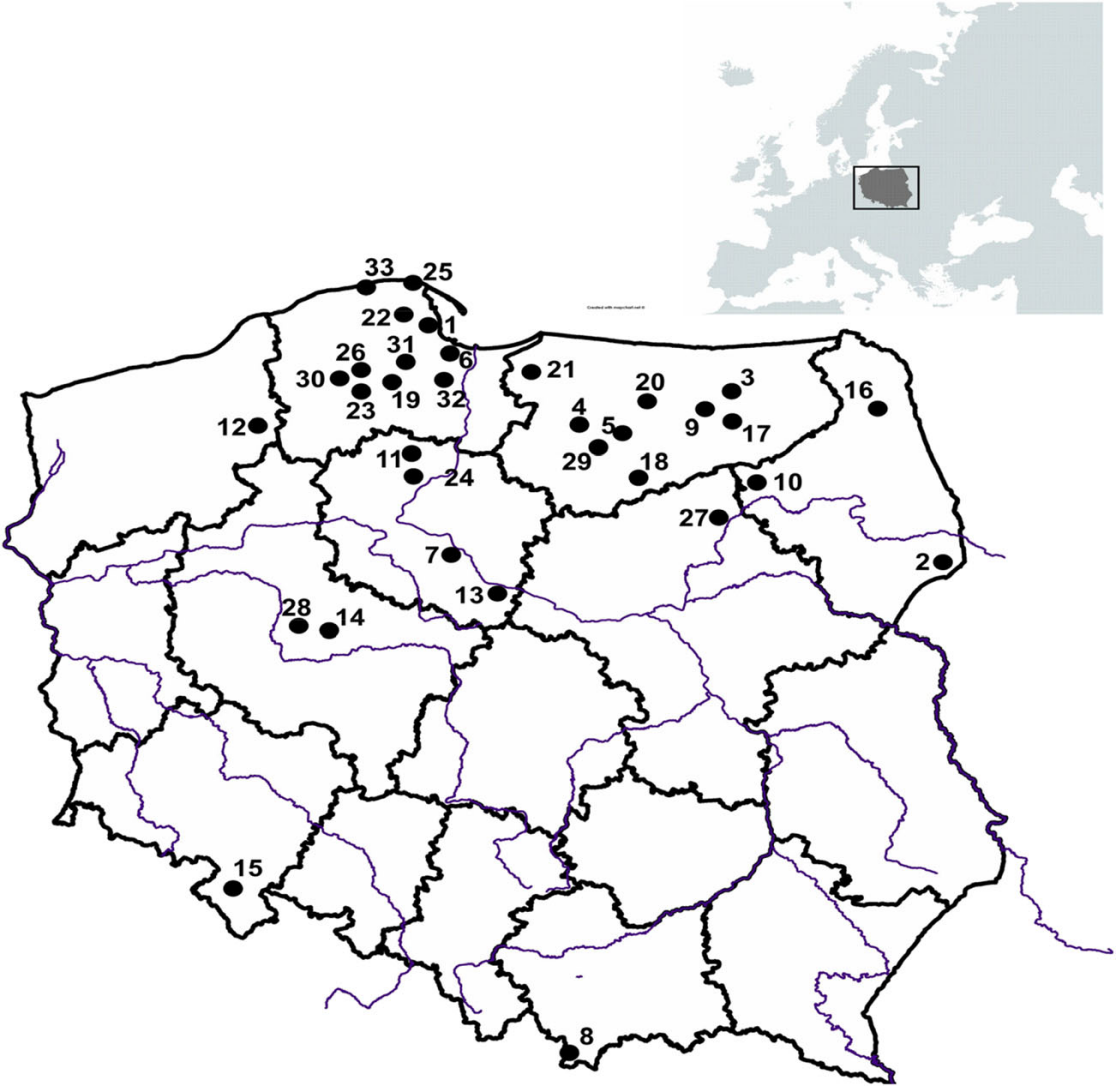
Location of the sampling sites (from 1 to 33) of *B. edulis* (site Id. shown in Table 1)

Samples of King Bolete (called also cep, porcini, etc.) *Boletus edulis* Fr. were collected from 33 locations across Poland in 15 of the 25 years during the period 1995–2019. Sampling locations are shown in Fig. 2, with a listing of the sites and year of sampling given in Table 1. Multiple specimens were collected and pooled (mean = 13, ranging from 2 to 40 specimens per pool), to obtain a composite sample representing each location (Table 1). In some years, multiple sites in different regions were sampled (e.g. nine locations in 2000). All of the fruiting bodies selected for study were in good condition and would be considered suitable for consumption (i.e. they were not infested by insects) or partially eaten by other animals. The maturity stage of the specimens ranged from young with white hymenophore to mature (large) with yellow hymenophore, but very young button stage mushrooms were not included. Directly after being picked, the fresh fruit bodies were cleaned from any visible plant, vegetation or soil debris with a plastic or ceramic knife and a sterile plastic brush, and the bottom part of stipe was cut-off.

**Table 1:** ^137^Caesium, ^40^Potassium (Bq kg^-1^ dry weight) and the nuclide cap to stipe activity concentration quotients (Q_C/S_) in *B. edulis* sampled in Poland during the period 1995–2019

Year of collection	Sampling region	Number per pool	Caps (whole)*	Stipes	Q_C/S_	Caps (whole)*	Stipes	Q_C/S_
			^137^Cs			^40^K		
1995	Tricity Landscape Park, Pomerania (1)	15	270 ± 15	160 ± 11	1.7	700 ± 250	400 ± 190	1.7
1998	Białowieża Primeval Forest, Podlasie (2)	15	10,000 ± 83	1200 ± 18	8.3	790 ± 120	500 ± 120	1.6
1998	Borecka Wilderness, Mazury (3)	3	660 ± 17	370 ± 9	1.8	600 ± 220	560 ± 110	1.1
1998	Łukta, Morąg, Piecki, Warmia (4)	24	(480 ± 9)			(620 ± 99)		
1999	Olsztyn region, Warmia (5)	15	(2100 ± 19)			(560 ± 79)		
1999	Sobieszewo Island, Pomerania (6)	9	1500 ± 27	780 ± 13	1.9	950 ± 180	590 ± 120	1.6
1999	Toruń forests, Kujawy (7)	15	(670 ± 7)			(510 ± 64)		
1999	Chochołowska Dale, Tatra Mountains (8)	12	(230 ± 5)			(760 ± 110)		
2000	Giżycko, Mazury (9)	21	740 ± 11	340 ± 5	2.2	770 ± 95	320 ± 68	2.4
2000	Wanacja, Kurpiowska Forest, Podlasie (10)	15	1300 ± 25	670 ± 21	1.9	760 ± 110	260 ± 180	2.9
2000	Tuchola Pinewoods, Pomerania (11)	6	(480 ± 6)			(590 ± 71)		
2000	Szczecinek, Rzecznica, Pomerania (12)	22	(570 ± 11)			(790 ± 120)		
2000	Goreń forests, Kujawy (13)	15	980 ± 14	530 ± 7	1.8	940 ± 110	410 ± 85	2.3
2000	Września, Wielkopolska (14)	3	(200 ± 6)			(590 ± 130)		
2000	Kłodzka Dale, Sudety Mountains (15)	10	5700 ± 5	3500 ± 3	1.6	900 ± 120	370 ± 90	2.4
2000	Augustów Primeval Forest, Suwalskie region (16)	16	(720 ± 8)			(670 ± 75)		
2000	Tuchola Pinewoods, Pomerania (11)	4	590 ± 27	360 ± 21	1.6	540 ± 350	230 ± 150	2.3
2001	Piska Wilderness, Mazury (17)	15	1000 ± 11	530 ± 10	1.9	610 ± 84	200 ± 95	3
2001	Puchałowo, Warmia (18)	15	(1900 ± 23)			(780 ± 130)		
2001	Kościerzyna forests, Pomerania (19)	26	970 ± 24	410 ± 12	2.4	760 ± 190	340 ± 150	2.2
2001	Pomerania (19) (caps, without hymenophore)^#^	12	940 ± 23	560 ± 21	1.7	800 ± 200	250 ± 150	3.2
2001	Kościerzyna forests, Pomerania (19) (hymenophore)	12	1200 ± 26			780 ± 180		
2001	Kościerzyna forests, Pomerania (19) (skin)	12	930 ± 34			3600 ± 400		
2002	Piska Wilderness, Szczytno, Mazury (17)	15	(770 ± 9)			620 ± 85		
2002	Kiwity, Warmia (20)	13	840 ± 15	460 ± 8	1.8	760 ± 150	490 ± 110	1.5
2002	Elbląg uplands, Pomerania (21)	7	(300 ± 9)			(350 ± 140)		
2003	Elbląg uplands, Pomerania (21)	7	(200 ± 5)			(410 ± 85)		
2003	Darżlubska Wilderness, Pomerania (22)	15	560 ± 11	260 ± 5	2.1	1100 ± 130	470 ± 91	2.3
2006	Tricity Landscape Park, Osowa, Pomerania (1)	15	(780 ± 17)			(670 ± 200)		
2006	Lipusz, Pomerania (23)	20	320 ± 4	190 ± 3	1.7	780 ± 71	370 ± 61	2.1
2006	Tuszynki, Pomerania (24)	6	(200 ± 8)			(730 ± 160)		
2007	Seashore Landscape Park, Pomerania (25)	16	300 ± 8	220 ± 5	1.4	630 ± 120	560 ± 110	1.1
2007	Mojusz, Pomerania (26)	11	(1400 ± 17)			(910 ± 130)		
2007	Olszewo-Borki/Lelis, Mazowsze (27)	15	(1000 ± 12)			(930 ± 100)		
2008	Porażyn, Wielkopolska (28)	13	(190 ± 4)			(520 ± 78)		
2008	Orzechowo/Olsztynek, Warmia (29)	10	1900 ± 39	1600 ± 41	1.2	680 ± 220	640 ± 270	1.1
2010	Parchowo, Pomerania (30)	15	500 ± 9	260 ± 4	1.9	730 ± 110	320 ± 76	2.3
2015	Pomlewo, Pomerania (31)	4	(15 ± 2)			(1600 ± 160)		
2015	Pomlewo, Pomerania (31)	40	51 ± 2	61 ± 2	0.84	850 ± 70	680 ± 60	1.2
2015	Darżlubska Wilderness, Pomerania (22)	15	(470 ± 9)			(760 ± 60)		
2016	Pomlewo, Pomerania (31)	3	(63 ± 2)			(840 ± 88)		
2016	Sobowidz, Pomerania (32)	6	26 ± 3	14 ± 1	1.9	910 ± 180	870 ± 82	1
2016	Lubiatowo, Pomerania (33)	23	(870 ± 39)			(870 ± 70)		
2016	Tuchola Pinewoods, Pomerania (11)	2	(81 ± 3)			(850 ± 130)		
2018	Pomlewo, Pomerania (31)	6	(53 ± 2)			(980 ± 74)		
2019	Pomlewo, Pomerania (31)	15	(25 ± 7)			(930 ± 190)		

^#^Deskinned; Q_C/S_ (quotient of cap to stipe activity concentration); *(data for whole fruiting bodies are in parentheses)

When not analysed as a whole, the fruiting bodies were separated into cap and stipe (stem supporting the cap) to get an insight into the physiological distribution of the nuclides within the mushroom. All samples were sliced into pieces using a plastic or ceramic knife and initially dried for 1 to 2 days at room temperature, followed by drying to constant mass in an electrically heated commercial fruit and vegetable dryer (model: MSG-01 dehydrator by MPM Product, Milanówek, Poland) at 65 °C. Individual samples were ground to a fine powder using a porcelain mortar and were then sealed in polyethylene bags. Individual bagged samples from a set were placed in larger plastic bags and stored sealed under dry and clean conditions in a large plastic container in a storage room until further analysis (Frankowska et al. 2010).

Before determination of the activity concentrations of radionuclides, the individual dried cap and stipe samples were pooled separately, packed into brand new polyethylene bags and stored sealed. ^134^Cs, ^137^Cs and ^40^K nuclide activities were measured, and the contents of total (stable) K were computed from the results of the ^40^K measurement, using the mean ^40^K activity concentration (range 27.33 to 31.31 Bq g^-1^) in natural K.

The methodology used to determine the nuclide activity in this study has been presented in detail in an earlier report (Falandysz et al. 2020). Briefly, a gamma spectrometer fitted with a coaxial high purity germanium (HPGe) detector was used to measure ^134^Cs, ^137^Cs and ^40^K activity. The instrument was calibrated with a multi-isotope standard using a fully validated method. Measurements were carried out on sample aliquots of 10–20 g of dehydrated fungal material that was lyophilised for 72 h before measurement. All measurements of ^137^Cs activity concentrations (^134^Cs was not detected in any sample) were decay corrected back to the day of collection.

Measurements were validated using a standard solution of gamma emitting isotopes, “code BW/Z-62/27/07”, produced by IBJ-Świerk, Otwock, Poland. This standard was used to prepare reference samples for spectrometer calibration. The background activity (80,000 s or 250,000 s) measured before analysis was subtracted (using the GENIE 2000 program) from the sample measurement.

## Results and discussion

### Contamination

Data on the occurrence of ^137^Cs and ^40^K in the whole fruiting bodies of *B. edulis* and its morphological parts (cap, hymenophore, skin, stipe) in this study have been detailed in Table 1. In Table 2, are summarised data available from literature on activity concentrations of ^137^Cs in *B. edulis* from Poland in 1984–1995. The data in Tables 1 and 2 are listed chronologically, and all concentrations were rounded to two significant figures. The rounding is a reasonable approach based on the accuracy of records as well as from the performance of the methodology in international calibration trials. It also allows better comparability with data from other laboratories.

**Table 2:** Activity concentration of ^137^Cs or ^137^Cs + ^134^Cs in *Boletus edulis* (Bq kg^-1^ dry weight; the amounts were rounded off to two significant figures) from Poland in 1984–1995, adapted

Localization, year and sample size (*n* = number of fruiting bodies)	^137^Cs or ^137^Cs + ^134^Cs	Reference
Rogóźno^#^, 1984; *n* = 1^#^	95	A
Rogóźno^#^, 1985; *n* = 1	100	A
1985; *n* = 1	600	B
Rogóźno^#^, 1986; *n* = 1	290	A
1986; *n* = 27	360^a^	B
Rogóźno^#^, 1987; *n* = 1	240	A
1987; *n* = 10	380^a^	B
near Gdańsk, 1987; *n* = 1	370	C
Rogóźno^#^, 1988; *n* = 1	440	A
1988; *n* = 8	900^a^	B
1989; *n* = 16	620^a^	B
1990; *n* = 33	750^a^	B
Augustowska Primeval Forest,1990; *n* = 4	1100	D
1991; *n* = 39^*^	710^a^	B
1995; *n* = 1	320	E
1995; *n* = 1	580	E

#Number of individual samples; *Composite sample; ^a^Assuming moisture content at 90%; ^#^550 km to the NPP in Chernobyl; *A* Bem et al. 1990; B Grabowski et al. 1994; *C* Korky and Kowalski, 1989; *D* Mietelski et al. 1994; *E* Calmet et al. 1998

Table 1 does not list the activity results measured for ^134^Cs as no activity resulting from this nuclide could be detected in the *B. edulis* samples in this study at the time of measurement (Table 1). Another study (Bem et al. 1990) which sampled *B. edulis* prior to the Chernobyl incident in Poland in 1985, at ca. 500 km north-west of the town of Chernobyl, was also unable to detect ^134^Cs. ^134^Cs has a much shorter half-life (2.06 years) in comparison to ^137^Cs (30.17 years) and can be used as a tracer of fresh emission and deposition/accumulation of radiocaesium from sources such as nuclear weapon testing or releases from nuclear power plant accidents such as those at Chernobyl and Fukushima, and also to follow the origin of ^137^Cs accumulated in environmental compartments.

^134^Cs has been found in *B. edulis* from locations that more or less closely neighbour the Chernobyl nuclear power plant, in Ukraine, Belarus and Russia, and also in mushrooms from more distant sites, including locations in Czechoslovakia, Austria and Finland, in 1986 or specimens from Germany during 1986–1987, Italy in 1986 and from France in 1995 and 1991–1997 (Battiston et al. 1989; Calmet et al. 1998; Elstner et al. 1987, 1989; Horyna and Řanda, 1988; Kirchner and Daillant, 1998; Marzano et al. 2001; Rantavaara, 1987; Teherani, 1988).

In Poland, ^134^Cs has not been detected in *B. edulis* samples that were collected at various distances from Chernobyl in the few months following the release of radioactivity in April 1986—both, during the mushrooming season of 1986, nor in the following year, 1987 (Bem et al. 1990; Grabowski et al. 1994) (Table 2). Contrarily, a positive detection of ^134^Cs was reported for a sample of *B. edulis* collected 4 years later from the Augustowska Primeval Forest (an area that in practice is the same as site No. 16 from this study, Fig. 2) in 1990 by Mietelski et al. (1994) (Table 2). ^134^Cs was also detected in two specimens of *B. edulis* (site details were not reported) from Poland in 1995 (Calmet et al. 1998) (Table 2). The detection of ^134^Cs in *B. edulis* from the Augustowska Primeval Forest in 1990 which is at the border with Belarus was analysed shortly after collection and may be explained by the relatively severe deposition (via sedimentation) of radiocaesium particles from the first plume emitted from the Chernobyl source (Fig. 1). However, depositions and the subsequent pollution of land were highly uneven for certain areas of Poland, which resulted in many local or regional hotspots (Fig. 1). At these hotspots, such depositions could have a high impact on the forested areas in general, and particularity on wild mushrooms and their local consumers. These anomalies provide a more general picture of the deposition profile even for the areas far away (up to − 1000 km) from the source (Fig. 1) (Bakken and Olsen, 1990; Mietelski et al. 2010).

The illustration of hotspots and deposition rates of radiocaesium on different areas in Poland (Fig. 1) is perhaps better mirrored in the results of ^137^Cs determinations in *B. edulis* in this study (Table 1) as well as through the reports on this nuclide in other mushrooms (Falandysz et al. 2015, 2016 and 2019; Mietelski et al. 1994 and 2010). Better resolution of the data reported on radionuclide deposition matters, particularly for the advice on consumption and health given to locals, including many mushroomers living in remote areas such as dispersed settlements and villages in the forested areas in Poland, or even worldwide.

In this study, ^137^Cs contamination of *B. edulis* was observed in all sets of fruiting bodies that were studied during the last 25 years in Poland, from 1995 to 2019. As mentioned, no ^134^Cs activity was detected in these samples collected 1995–2014 and examined in 2015, nor in the later samples collected from 2015 to 2019 which were examined almost directly after collection. It is not surprising that the mushrooms collected over a 25 years’ time-span revealed a heterogeneous (uneven) spatial distribution of ^137^Cs activity concentrations in the whole fruiting bodies, e.g. in 2000, activity ranged from 200 ± 6 Bq kg^-1^ dw at the Września site in Wielkopolska to 720 ± 8 Bq kg^-1^ dw at the Augustów Primeval Forest (Suwalskie region), and in fungal caps, from 740 ± 11 Bq kg^-1^ dw at the Giżycko (Mazury) site to 5700 ± 5 Bq kg^-1^ dw in Kłodzka Dale (Sudety mountains). The earliest *B. edulis* sample investigated in this study that was collected in 1995 from the Tricity Landscape Park (a forested complex lying immediately west to the centres of the cities of Gdańsk, Sopot and Gdynia) showed ^137^Cs concentration activity in caps of 270 ± 15 Bq kg^-1^ dw. The specimens collected in 1998 from the Białowieża Primeval Forest in Podlasie (which was shown to be a possible hotspot, from the current data) showed ^137^Cs activity in the caps at 10,000 ± 83 Bq kg^-1^ dw, but quite distinctly from other sites showed much lower activity in the stipes (Q_C/S_ 8.3). The cap to stipe quotient (Q_C/S_) for ^137^Cs fruiting bodies in all other samples in this study was in the range of 0.84 to 2.2 (Table 1).

Relatively higher ^137^Cs activity in *B. edulis* from several sites in Poland in particular years were noted in samples from the Białowieża Primeval Forest and Olsztyn regions (1998), Wanacja and Kłodzka Dale (2000), the Piska Wilderness and Puchałowo (2001), Olszewo-Borki/Lelis (2007) and Orzechowo/Olsztynek (2008). This is likely to be due to higher levels of depositions to the soils in these areas (Fig. 1). However, it is unclear as to whether the *B. edulis* collected from Mojusz (Pomerania), showing activity concentration of ^137^Cs of 1400 ± 17 Bq kg^-1^ dw, or from the Sudety mountains (5700 ± 5 Bq kg^-1^ dw ) which may be considered substantially contaminated, could have resulted from possible microscale events of wet deposition. A number of these higher activity levels were seen in sampled locations in eastern parts of Poland (apart from that in the Sudety mountains), which is consistent with the patterns of projected depositions (Fig. 1) following the Chernobyl incident.

### Trends in contamination

**Fig. 3:**
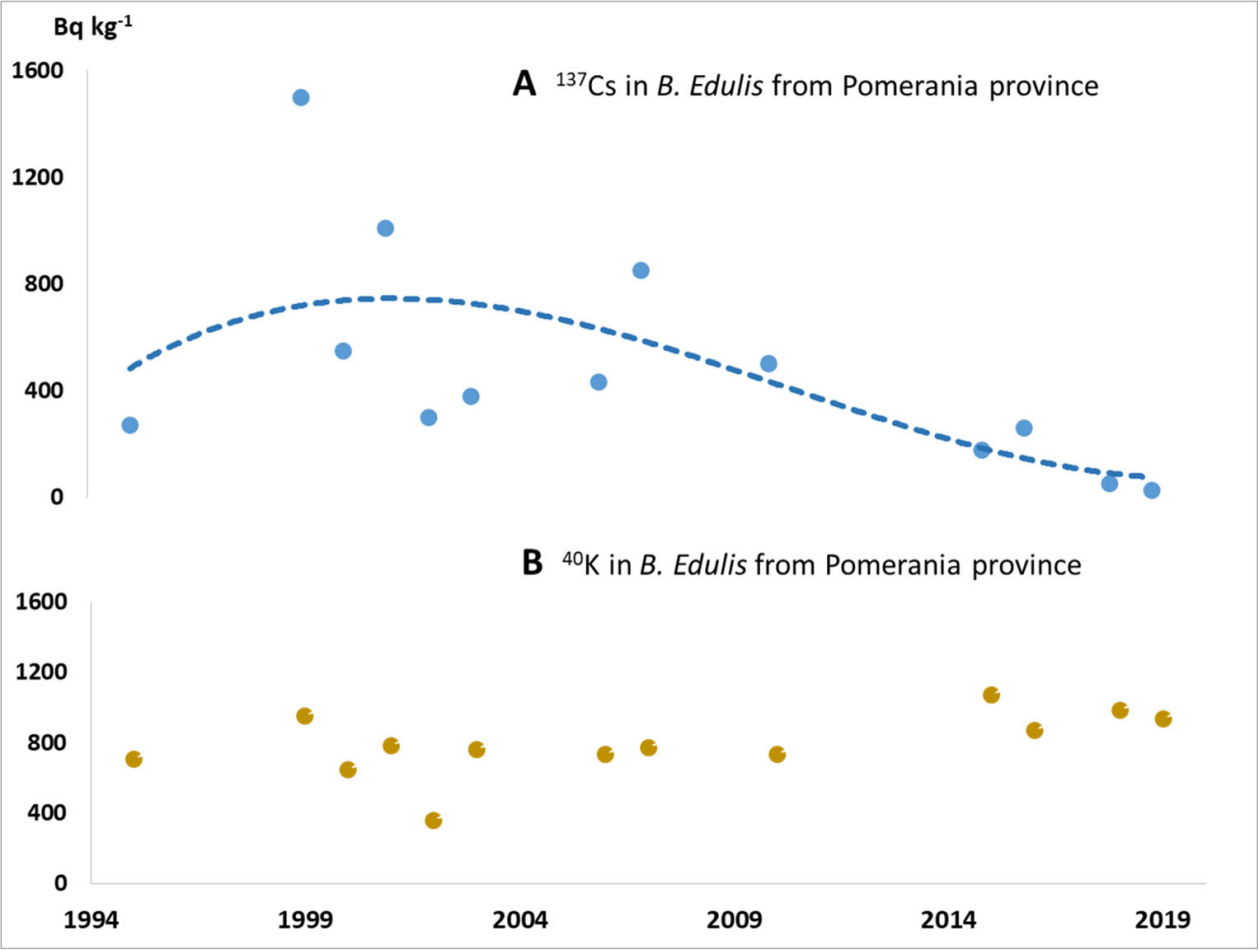
Mean activity concentrations (Bq kg^-1^ dw) per year of sampling. **a** ^137^Cs showing indicative trend (dashed line) and **b** ^40^K activity concentrations in *B. edulis* samples from Pomerania province 1995–2019

Collectively, these results reflect the complex fallout, deposition and soil accumulation patterns of ^137^Cs and indicate the extent of sampling and investigation that would be required to accurately characterise areas. Although the coastal province of Pomerania was perceived as being low to moderately impacted by the Chernobyl fallout (Fig. 1), the effect on mushroom activity concentrations was clearly noticeable. It is fortunate that a large proportion of the current study samples were taken from this region as this allows examination of the temporal dispersion of the mean ^137^Cs concentrations in *B. edulis* from this province, as shown in Fig. 3a. Higher activity levels are evident in the period between 10 and 20 years after the Chernobyl incident (1986) which then slowly declines towards the present time. The data are however too few and variable to distinguish a declining trend. By contrast, the ^40^K activity which is naturally present as part of the total potassium content of mushrooms does not appear to vary too greatly in the same mushrooms over this period (Fig. 3b). The activity concentrations (range, 190–10,000 Bq kg^-1^ dw) recorded in this study during 1995–2010 were, on average, higher than those reported in *B. edulis* (240–2300 Bq kg^-1^ dw) by other authors for fruiting bodies collected across the country over an earlier period, from 1984 to 1994 (Bem et al. 1990; Grabowski et al., 1994; Korky and Kowalski, 1989; Mietelski et al. 1994; Calmet et al. 1998). Collectively, both sets of data agree with the general hypothesis (Mietelski et al. 2010) that deposited ^137^Cs permeates slowly over many years, to deeper soil horizons where the mycelial network of this mycorrhizal fungus proliferates. The resulting delay in the uptake of ^137^Cs shows that (apart from hotspots) higher contamination of the fruiting bodies appears to have occurred between 10 and 20 years after the incident.

**Fig. 4:**
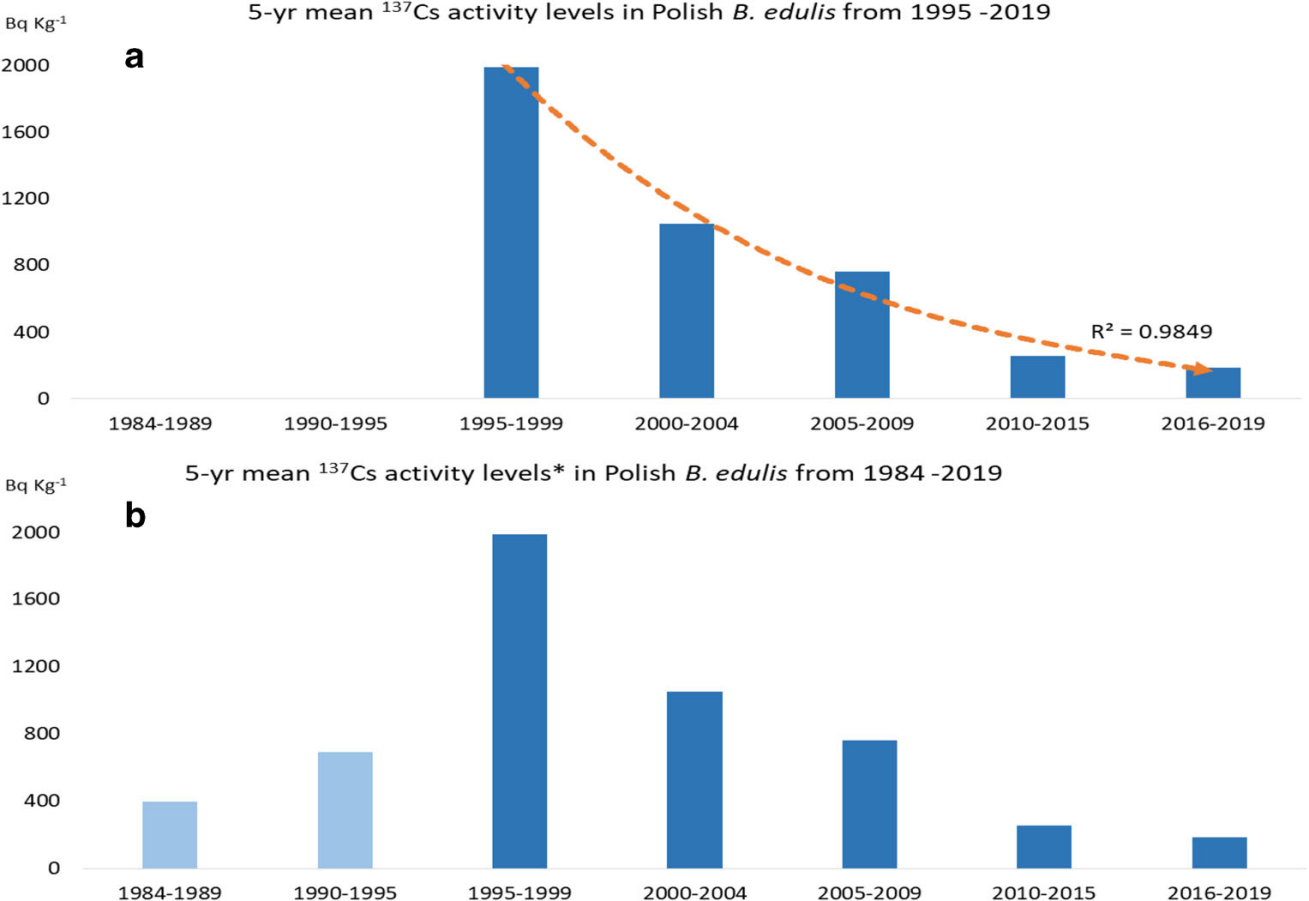
Mean^137^Cs activity concentrations (Bq kg^-1^ dw) in *B.* *edulis* in Poland over the period 1984–2019, showing that the decline in concentrations since maximum activity was noted in 1995–1999 (excludes hotspots)

Morphologically, the bulk of the mycelial biomass of *B. edulis* is located somewhat deeper in the forest soil horizon in comparison to many other species. However, following deposition, radiocaesium is substantially retained in the upper soil layers in forests (including accumulation in mycelium, mycelial cords (rhizomorphs) and hypogeous (underground) basidiocarps)) and migrates slowly to lower levels of undisturbed soil (Clouvas et al. 2007; Falandysz et al. 2018; Mietelski et al. 2010; Steiner and Fielitz, 2009). Because of its shorter half-life, ^134^Cs is depleted to a far greater extent than ^137^Cs during the time taken to permeate to the deeper soil layers where the mycelial biomass proliferates. There is therefore a time lapse before the resulting enrichment of these layers with ^137^Cs. This delay in the availability of ^137^Cs at higher levels, following deposition and its subsequent absorption and translocation to emerging fruiting bodies, results in the highest ^137^Cs activity levels in *B. edulis* occurring later than in mushrooms with mycelia that dwell largely in the shallow layers of soil or in the decaying leaf litter (Falandysz et al. 2018; Mietelski et al. 2010; Řanda et al. 1990). Figure 4a shows the full dataset (*n* = 46 sets of observations, at all locations) from this study. Figure 4b includes literature data (Bem et al. 1990; Grabowski et al. 1994; Korky and Kowalski, 1989; Mietelski et al. 1994; Calmet et al. 1998) on ^137^Cs in *B. edulis* from Polish locations for the years from 1984 to 1995. In order to minimise the temporal and spatial variations from single years and different locations, the averaged data for the 5-year intervals was plotted in both Fig. 4 a and b. The maximum ^137^Cs activity is seen in the years from 1995 to 2009, and the decline in activity to the present time is evident in Fig. 4a. The trend-line (*r* = 0.984) underlines the significance of the decline over this period (Pearson correlation value of − 0.96). However, it is the earlier reported data from 1984 to 1995 (Bem et al. 1990; Grabowski et al. 1994; Korky and Kowalski, 1989; Mietelski et al. 1994; Calmet et al. 1998) that supports the hypothesis of delayed permeation to lower soil layers (Fig. 3b), as the ^137^Cs activity in this ectomycorrhizal fungus with relatively deep mycelial networks was considerably lower in the years immediately after the Chernobyl accident, but showed the highest mean values during 1995–1999, i.e. almost a decade after the accident.

### Other factors that influence the uptake of ^137^Cs

Another important and relevant reason for the observed concentrations is the natural ability of many species of mushrooms to bio-concentrate stable caesium (^133^Cs), as well as its abundance in soils (Klán et al. 1988; Řanda et al. 1990). Another mineral constituent that plays a part in this bio-concentration process appears to be rubidium (Rb), which is the most physically alike element to Cs (Falandysz and Borovička 2013; Vinichuk et al. 2010). *B. edulis* is good accumulator of ^133^Cs with a bio-concentration factor (BCF) of 6.6 ± 1.0, but it is a far more efficient accumulator of rubidium (Rb) which shows a BCF of 345 ± 2 (Řanda et al. 1990).

Apart from the post-Chernobyl bio-concentration of radiocaesium that was observed in the late 1990s and subsequent years, it is important to note that mushrooms including *B. edulis* in Europe were already bio-concentrating ^137^Cs from pre-Chernobyl fallout sources. For example, *B. edulis* from the Reggio-Emilia in Italy in the early 1990s were found to accumulate from 20 to 46% of ^137^Cs deposited in this region (Cocchi et al. 2017) from the global fallout following the detonation of nuclear weapons during the middle of the last century. The time lapse of around a quarter of a century since the accident which was the last known release of radioactivity in Europe (minor incidents have been reported, e.g. at Paks, Hungary, at Forsmark in Sweden, but the impacts from these are likely to be local) is a significant period relative to the half-life of ^137^Cs. Additionally, the biological half-live of radiocaesium accumulated in *B. edulis*, like in other wild growing mushrooms, is shorter (as can be derived also from data in Table 1 and Fig. 4) in comparison to the physical half-life, due to lower migration of ^137^Cs with depth and also the parallel process of dilution with greater depths.

Due to the source of their nutritional requirements or other growth and habitat characteristics, macromycetes (fungi) are classified basically as saprobic, mutualists (symbiotic) and parasites. Ectomycorrhizal symbionts (mutual relationship with plant roots) usually have a greater potential to bio-concentrate radiocaesium than other types although a mechanism that explains this potential has still not been completely resolved (Falandysz and Borovička 2013). Radiocaesium is absorbed by mushrooms from soil together with the stable ^133^Cs (Řanda et al. 1990; Yoshida et al. 2004). Thus, the status of ^133^Cs in mushrooms and in the associated soil as well of co-absorbed Rb can also influence the species-dependent differences in predilection to accumulate radiocaesium, but this aspect has been little studied so far.

Following deposition, radiocaesium can be taken up rapidly by the mycelia of some fungal species and incorporated into emerging fruiting bodies. This depends on the depth of the mycelial network as well as the fruiting bodies which emerge underground. Rapid absorption of a portion of radiocaesium from fresh deposition in undisturbed forests is possible in some cases. It has been documented (Teramage et al. 2014) that a proportion of deposited radiocaesium could be transferred to lower layers of the soil horizon quickly, carried by rainwater through large pores in topsoil layers in undisturbed forests. A rapid and elevated accumulation of radiocaesium has been observed directly after the Chernobyl accident in species like *Laccaria amethistina* where the mycelia feeds largely in the fermentation layer of forest litter, while mycorrhizal species collected from the same sites such as *Cortinarius caperatus* (previous name *Rozites caperata*) and *Imleria badia* (previous name *Xerocomus badius*) which are known to be efficient accumulators of this nuclide were much lower in ^134/137^Cs in 1986 (Stijve and Poretti, 1990). Clearly, many factors play a part in the efficiency of the bio-concentration process such as the absorption and strong retention of radiocaesium by fermenting and humified layers of forest soil, which are rich in detritus, microbes, invertebrates, plant and mycelial networks, and the biogeochemical cycling of the nuclide in view of its physical persistence. Additionally, some forest plants and animals that disturb the soil assist the process by moving nuclides from deeper layers of soil up to the surface. This redistribution combined with fresh litter fall depositions can together result in a long-lasting contamination by this nuclide in some mushroom species.

### ^40^K activity

*B. edulis* like other ectomycorrhizal mushrooms is rich in potassium (K), which is efficiently bio-concentrated by this species from the soil background. It is usually found in greater concentration in the caps (20,000 ± 4,000 to 38,000 ± 2,000 mg kg^-1^ dw) than in the stipes (12,000 ± 2000 to 21,000 ± 7000 mg kg^-1^ dw) of fruiting bodies (Falandysz et al. 2011; Frankowska et al. 2010). A very small proportion of this natural K consists of a radioactive counterpart. Thus, *B. edulis* like other mushrooms always emits some natural background radioactivity due to the accumulation of the long-lived ^40^K (half-life of 1.248 × 10^9^ years) that emits high energy gamma-radiation and also some beta-radiation. The uptake of K is related to the essential biological function of this element in mushrooms*.* The concentrations in fruiting bodies are highly regulated by different species due to the vital role of K in hydration (fresh *B. edulis* has around 90% moisture content) and other life functions of fungi.

*B. edulis* showed ^40^K activity concentrations in the whole fruiting bodies in the range from 350 ± 140 Bq kg^-1^ dw at the Elbląg upland site in Pomerania in 2002 to 1600 ± 160 Bq kg^-1^ dw, in specimens collected from the site at Pomlewo in Pomerania during 2015. The distribution of this nuclide between caps and stipes (Q_C/S_ in the range 1.0 to 3.2; see Table 1) followed a general tendency reported for total K (Falandysz et al. 2008 and 2011; Frankowska et al. 2010). Curiously, *B. edulis* showed the highest activity concentration of ^40^K of 3600 ± 400 Bq kg^-1^ dw in the skin of the cap, unlike ^137^Cs, which was highest in the hymenophore of the same specimens, i.e. at 1200 ± 26 Bq kg^-1^ dw (Table 1).

### Radiocaesium intake from the consumption of *B. edulis*

The health risk from the dietary intake of radiocaesium is well recognised, and the level of this contaminant in foods, particularly susceptible foods such as wild mushrooms, is controlled in many countries. The tolerance limit in the European Union (and Switzerland) is 600 Bq kg^-1^ wet weight (ww) in food for adults and 370 Bq kg^-1^ ww in food for infants (EC, 2000). In the Codex Alimentarius, the intervention level defined for fresh foods is 1000 Bq kg^-1^ (FAO/WHO, 1991). The specific activity limit for radiocaesium in mushrooms in Belarus is 370 Bq kg^-1^ ww (Bulko et al. 2014). In view of the range of limits reported, dried *B. edulis* sampled over the years at many sites have exceeded some of these tolerances. However, assuming the moisture content of mushrooms at 90%, only the caps of specimens collected in 1998 from the permitted area of the Białowieża Primeval Forest which borders Belarus and has a relatively closer proximity to Chernobyl showed elevated activity concentration of ^137^Cs above these limits, i.e. of 1000 Bq kg^-1^fresh weight (Table 1).

In most cases, the consumption of fresh or dried mushrooms requires some form of culinary processing based on the recipe or type of cooking used. This processing usually results in the loss of a portion of the accumulated ^134/137^Cs because of leaching into discarded water or oil phases (rinsing, blanching, boiling, frying) (Daillant et al. 2013; Saba and Falandysz 2021; Steinhauser and Steinhauser 2016). On the other hand, the partial dehydration of fruiting bodies during braising in fat, deep-oil stir-frying in a wok or frying in a flat pan increases the whole weight radiocaesium activity in the final dishes (fried or braised mushrooms) by up to two-fold when compared to the fresh (wet) substrate mushrooms (Falandysz et al. 2020 and 2021). This has been observed for mature specimens, but recent studies have found that button stage *B. edulis* and *Amanita muscaria* can be highly elevated (above ten-fold) in radiocaesium content when compared to young and mature specimens. Clearly, this would result in much higher intake rates of this nuclide for those consumers who prefer button stage *B. edulis* (Falandysz et al. 2019 and 2021). Thus, considering that edible wild mushrooms are an important source of radiocaesium intake for some populations (Řanda et al. 1990; Rantavaara, 1987), with tolerances specified for dietary intake levels in foods, a subset of consumers (those who prefer button stage *B. edulis*, which are highly prized by many mushroomers or specific mushroom meals that call for button stage produce) are likely to have a higher level of radiocaesium exposure. This is clearly dependent on factors such as the quantity and rate of consumption, level of contamination and proximity to sources, and more detailed exposure scenarios would be required to assess the resulting health risk.

## Conclusions

The popular and prized *B. edulis* mushroom appears to be a good bio-indicator of the distribution of radiocaesium in forested and woodland areas of the Polish environment. In this study, the chronology of collection and analysis of this species from different locations dates back over 25 years from 1995 to the present time. The data show that the contamination was characterised mainly by ^137^Cs activity, although some studies have noted some ^134^Cs activity earlier in the period, which is consistent with its detection in the immediate aftermath of nuclear accidents such as the Chernobyl incident in 1986.

Although the surface of fruiting bodies may be contaminated directly during episodic microdeposition incidents resulting from the projected fallout plumes following the Chernobyl incident, the uptake of ^137^Cs by *B. edulis* in its natural habitats in forested areas is related to its morphology and the relatively greater depth to which its mycelial network penetrates the soil horizon. This results in a delay in the uptake of ^137^Cs in this species arising from the time required for permeation to the required depths, following deposition on the forest floor. The decline towards lower activity levels in the most recent samples suggests that as far as ^137^Cs contamination in *B. edulis* is concerned, the present time is a relatively “cleaner period” since the testing of nuclear weapons during the middle of the last century and the Chernobyl incident.

Foragers and recreational mushroom pickers who consume this species are undoubtedly exposed to ^137^Cs activity, although activity levels in the sampled mushrooms suggest that serious breaches of regulated levels were uncommon and most likely related to the Chernobyl incident. The variety of exposure scenarios including sampling location, level of contamination, amount consumed and the type of culinary processing are complex would perhaps form part of a further study.

## Data Availability

Not applicable.
